# Nuclear positioning in muscle development and disease

**DOI:** 10.3389/fphys.2013.00363

**Published:** 2013-12-12

**Authors:** Eric S. Folker, Mary K. Baylies

**Affiliations:** ^1^Department of Biology, Boston CollegeChestnut Hill, MA, USA; ^2^Department of Developmental Biology, Sloan-Kettering InstituteNew York, NY, USA

**Keywords:** Nuclear movement, muscle disease, nucleoskeleton, cytoskeleton

## Abstract

Muscle disease as a group is characterized by muscle weakness, muscle loss, and impaired muscle function. Although the phenotype is the same, the underlying cellular pathologies, and the molecular causes of these pathologies, are diverse. One common feature of many muscle disorders is the mispositioning of myonuclei. In unaffected individuals, myonuclei are spaced throughout the periphery of the muscle fiber such that the distance between nuclei is maximized. However, in diseased muscles, the nuclei are often clustered within the center of the muscle cell. Although this phenotype has been acknowledged for several decades, it is often ignored as a contributor to muscle weakness. Rather, these nuclei are taken only as a sign of muscle repair. Here we review the evidence that mispositioned myonuclei are not merely a symptom of muscle disease but also a cause. Additionally, we review the working models for how myonuclei move from two different perspectives: from that of the nuclei and from that of the cytoskeleton. We further compare and contrast these mechanisms with the mechanisms of nuclear movement in other cell types both to draw general themes for nuclear movement and to identify muscle-specific considerations. Finally, we focus on factors that can be linked to muscle disease and find that genes that regulate myonuclear movement and positioning have been linked to muscular dystrophy. Although the cause-effect relationship is largely speculative, recent data indicate that the position of nuclei should no longer be considered only a means to diagnose muscle disease.

## History

Myofibers are the cellular units of mature skeletal muscles. The structure of myofibers, and the basic principles that govern the development of myofibers, are conserved from *Drosophila* to humans. Skeletal muscle accounts for nearly 50% of adult body mass, and the organization of the myofibers is repetitive and striking. This repetitive structure is most notably illustrated by the myofibril network, the linear and repetitive arrangement of sarcomeres and associated proteins that enable muscle contraction. The myofibril network of skeletal muscle garnered much early attention and has been studied in detail since the early 1940s, when Ramsey and Street published their observations that the length of the sarcomere corresponded to the physical output of the muscle (Ramsey and Street, 1940). With improved electron microscopy techniques to better understand subcellular organization, the structure of the myofibrils was examined in more depth, culminating in development of the sarcomeric sliding filament model described in 1954 (Huxley and Hanson, 1954; Huxley and Niedergerke, 1954). Importantly, work in the field of muscle biology maintained its focus on correlating the structure of the muscle with the function, or physical output, of the muscle cell. Moving forward, the feature that the functional output of muscle can be easily assessed makes muscle an ideal tissue in which to understand additional aspects of cellular structure and organization and how they impact function.

With the contractile myofibrillary network described, and the development of more sophisticated imaging techniques, further definition of the myofiber structure and how that structure impacts function has gained traction. Coincident with the ability to more precisely examine muscle structure, advancements in sequencing and gene identification have made it evident that sarcomere assembly and myofibril organization are not sufficient for full muscle function. In fact, many mutations that cause muscle disease do not appear to directly affect sarcomere structure. For example, Emery-Dreifuss Muscular Dystrophy (EDMD) is characterized by progressive muscle weakness, but the genes that are mutated in patients with EDMD encode proteins that localize to the nucleus rather than the sarcomere. Furthermore, at least a subset of EDMD causing mutations do not impact the assembly of the sarcomere (Gueneau et al., 2009). This makes clear that sarcomere assembly on its own is not sufficient for muscle cells to generate maximal force and indicates that additional aspects of cellular organization impact muscle physiology and likely underlie many muscle diseases. Thus, to fully understand general muscle biology, and muscle disease pathogenesis specifically, we must determine how muscle cells become organized and the relative contributions of each aspect of organization to muscle function.

Like all eukaryotic cells, myofibers require several organelles that compartmentalize different cellular functions. For example, mitochondria compartmentalize energy production, the nuclei compartmentalize gene regulation, the sarcoplasmic reticulum compartmentalizes calcium storage and release, and the Golgi apparatus compartmentalizes protein sorting. Each of these organelles is essential to proper muscle function. This fact is illustrated by the identification of mutations in genes related to each organelle that cause muscle disease (Cohen et al., 2013; Gazzerro et al., 2013; Schreiber and Kennedy, 2013). Although the metabolic importance of muscle has been recognized for decades, and significant information regarding the relationship between mutations in metabolic enzymes and muscle disease exists (Muntoni et al., 2011; Bonaldo and Sandri, 2013), the role of general muscle architecture in muscle function is less clear. Little is known regarding the aspects of organization that are essential, how each organelle contributes to muscle function, and whether the positioning of different organelles are linked or occur independently.

These are overarching questions that will require years of work to understand as only recently have researchers begun studying the positioning of organelles in muscle. This review will focus on the organization of nuclei within the myofiber. Specifically, we will explore the mechanisms by which nuclei are positioned, and the evidence that the precise positioning of nuclei is essential for proper muscle function.

## Nuclear positioning in muscle

Nuclei in muscle are positioned at the periphery of each myofiber. Furthermore, these peripheral nuclei are positioned to maximize the distance between adjacent nuclei (Bruusgaard et al., 2003). Although it is not known why nuclei are positioned in this way, there are intuitive and compelling possibilities to explain both aspects of nuclear position. The myodomains theory states that each nucleus nourishes a discrete portion of the muscle (Pavlath et al., 1989) and provides a logical explanation for the maximizing of internuclear distances. If nuclei were clustered rather than spaced evenly, different regions of the muscle would lack the transcription and translation necessary to maintain the myofiber. Regarding the positioning of the nuclei at the periphery of the myofiber, rather than within the myofiber, it is intuitive that nuclei in the center of the myofiber could act as physical obstacles to contraction and therefore impede muscle output. Alternatively, maintaining nuclei at the muscle periphery may be a means to protect nuclei from the force of contraction that they would need to withstand in the central portion of the muscle. Importantly, these options are not mutually exclusive.

Consistent with these potential functions for myonuclear positioning, biopsies of the muscles from patients with several different muscle disorders display large numbers of myofibers with centrally positioned nuclei (>25% compared to <3% in unaffected individuals). Mispositioned nuclei were originally noted with respect to muscle disease by Dr. Spiro (Spiro et al., 1966) regarding a patient with Myotubular Myopathy, one of a subset of muscle diseases that would become collectively referred to as Central Nuclear Myopathies (CNM). However, centrally positioned nuclei are not unique to CNM and have been noted, and are prominent, in many distinct muscle disorders. Moreover, central nuclei have been routinely used for nearly 50 years as a pathological marker for differentiating muscle disorders from neurological disorders (Dubowitz et al., 2007). Indeed, muscle biopsies from patients with most muscle disorders, including relatively common disorders such as Duchenne Muscular Dystrophy (DMD) (Wang et al., 2000), Becker Muscular Dystrophy (BMD), and EDMD (Gueneau et al., 2009), show nuclei prominently within the center of individual muscle fibers.

However, despite the prevalence of centrally positioned nuclei in the myofibers of patients suffering from disparate muscle diseases, the importance of nuclear positioning to disease pathogenesis and muscle weakness is not clear. Moreover, there is little to be found in the scientific literature exploring the role of nuclear positioning in muscle function or disease. This is in part explained by the prevailing hypothesis that is used to explain centrally positioned nuclei: central nuclei are considered to be merely a marker of ongoing myofiber repair. This assumption is well supported by the general mechanisms of muscle development and repair during which all muscle nuclei undergo at least three dramatic movements.

**Figure 1:**
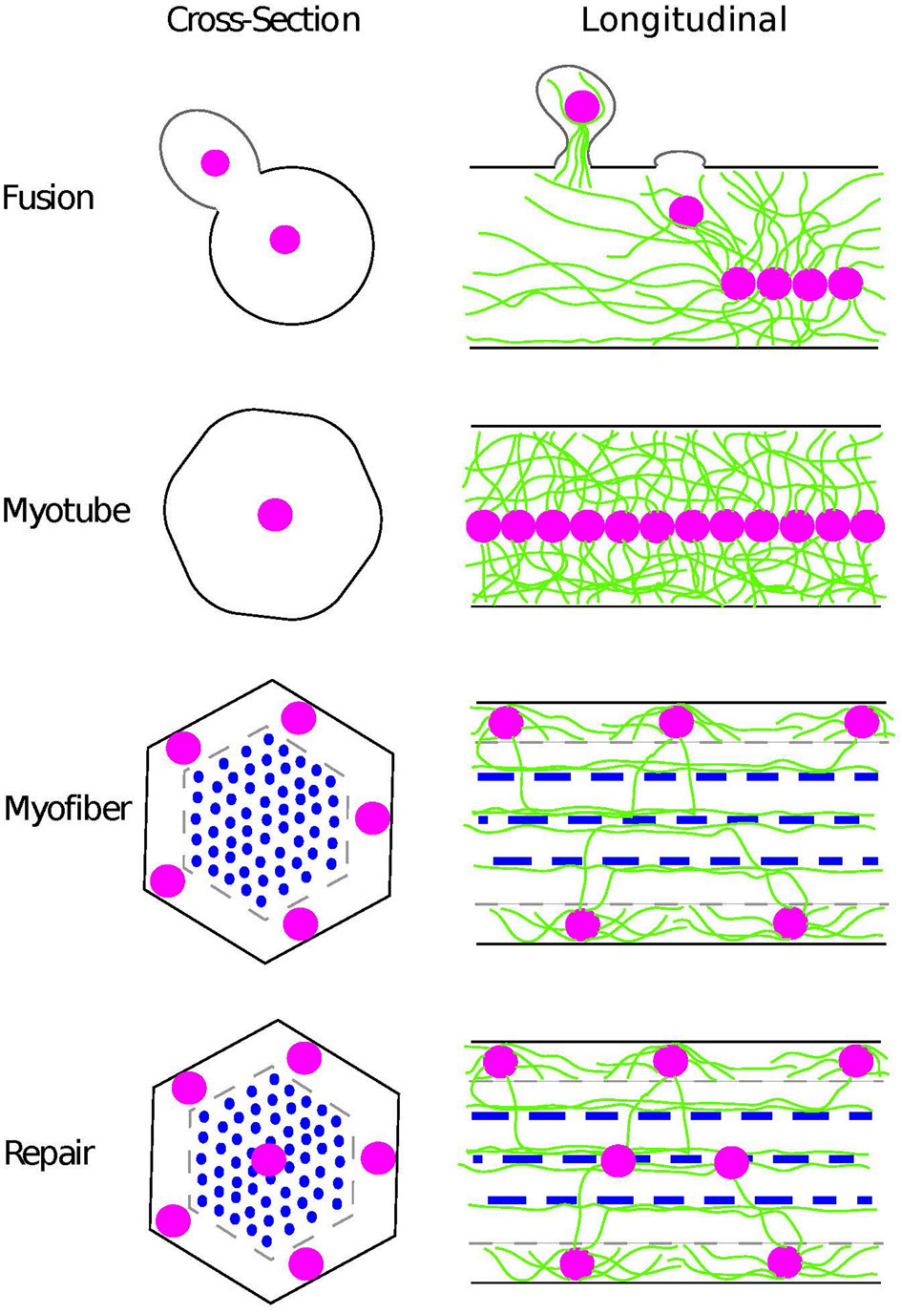
**Position of nuclei during muscle development as seen in cross-section (left) and longitudinal samples (right)**. As new nuclei (pink) are incorporated from myoblasts during fusion, they are rapidly moved to the center of the myotube by a process that requires the microtubule cytoskeleton (green). Thus, in the myotube, the nuclei are aligned in the center of the cell. As the myotube matures into a myofiber with the assembly of the sarcomere (blue), the nuclei move to the periphery of the muscle and reside directly above the sarcolemna (gray) and space to maximize the internuclear distance. Coincident with these nuclear movements, the microtubule cytoskeleton becomes highly ordered. Microtubules are nucleated at or near the nuclear envelope with some overlap of microtubules emanating from adjacent nuclei. Additionally, microtubules extend to the sarcomeres and run parallel to these highly ordered actin-myosin based structures. During repair, newly incorporated nuclei undergo movements similar to the movements of nuclei in the developing muscle. New nuclei are incorporated into the myofiber as myotubes fuse with the myofiber. The newly incorporated nuclei move to the center of the myofiber before moving out to the myofiber periphery in two separate microtubule-dependent processes.

Multinucleate muscle fibers form from the fusion of mononucleated myoblasts rather than through nuclear divisions in the absence of cytokinesis as was once thought (Capers, 1960). Upon fusion, each newly incorporated nucleus is actively moved to the center of the immature myotube (Kelly and Zacks, 1969; Cadot et al., 2012) (Figure 1). Following many fusion events, the myotube will mature into a myofiber. Historically, this maturation process is identified by the development of a dense myofibril network throughout the cell. However, this maturation process also correlates with the second type of nuclear movement during which nuclei are moved from the center of the myofiber to the periphery (Capers, 1960) and the third movement in which the distance between adjacent nuclei is maximized (Bruusgaard et al., 2003) (Figure 1). It is not clear whether the movement of the nuclei to the periphery and the assembly of the myofibril network are functionally linked and/or whether one process is dependent on the other. Yet, the coincident nature of these two events and the prevalence of aberrant nuclear positioning in individuals with muscle disease, suggest that the peripheral localization of nuclei and the maximizing of internuclear distance are important factors in muscle development.

Following the movement of nuclei to the muscle periphery, a small subset of muscle nuclei will undergo an additional movement. These myonuclei can move as either individuals or as clusters to the Neuromuscular Junction (NMJ) and stably localize there as clusters of between 3 and 8 nuclei (Englander and Rubin, 1987). This last movement to the NMJ is an active process, and these nuclei have unique transcriptional profiles and different levels of nuclear membrane proteins compared to the majority of the muscle nuclei (Sanes et al., 1991; Moscoso et al., 1995). Furthermore, it has been demonstrated that the positioning of these nuclei is essential for synaptic transmission (Jevsek et al., 2006) and that the absence of nuclei clustered at the NMJ correlate with neuro-muscular disease (Grady et al., 2005; Zhang et al., 2007).

Similar nuclear movements are seen during myofiber repair (Figure 1). First, activated satellite cells fuse with the damaged myofiber (Yin et al., 2013). However, rather than maintaining its position at the myofiber periphery where it fused, a newly incorporated nucleus is moved to the center of the myofiber before being moved back out to the cell periphery (Dubowitz et al., 2007). The reason for these long-range nuclear movements is not known. However, cross-sectional analysis reveals that many more myofibers will have centrally positioned nuclei when a muscle is undergoing repair compared to steady-state muscles. Thus, centrally positioned nuclei provide an easy assay to determine which myofibers are undergoing repair in response to either disease or physical insult (Dubowitz et al., 2007).

For all of these reasons, it has been presumed that centrally positioned nuclei are a consequence of continual myofiber repair in patients with muscle disease. Therefore, the possibility that mispositioned nuclei contribute to muscle weakness and disease have been ignored. However, that both nuclear movements are maintained in already mature myofibers suggests that there is a biological necessity to these movements. Significant energy is spent moving nuclei to the center of the myofiber and back to the periphery indicating that nuclear movement in muscle is necessary for proper muscle function. It is therefore essential to understand the mechanisms that drive these nuclear movements and the biological significance of these nuclear movements to fully understand and treat muscle disease.

Furthermore, many genes that are mutated in patients with muscle disease encode proteins that localize to the nucleus. The first identified proteins that localize to the nucleus and cause muscle disease have known roles in regulating gene expression (Maraldi et al., 2002; Tsukahara et al., 2002). Therefore, the initial, and still enticing, hypothesis was that muscle diseases associated with these mutations resulted from aberrant gene regulation. However, proteins that localize exclusively in the outer nuclear envelope and regulate the interactions between the nucleus and the cytoskeleton have recently been identified as mutated in patients with muscle disease (Wheeler et al., 2007; Zhang et al., 2007; Puckelwartz et al., 2009). Because these genes do not directly interact with the genome, these data raise the possibility that the nucleus may have a role in muscle development and function independent of its general role in gene regulation and might suggest a role for nucleus-cytoskeleton interactions and nuclear positioning in muscle development and disease pathogenesis.

We will review the mechanisms of nuclear positioning, specifically in muscle, from the perspective of both the nucleus and the cytoskeleton. Although we will discuss the mechanisms of nuclear movement in broad strokes, we will further focus the discussion toward genes known to be mutated in patients with muscle disease.

## The nucleus

It is intuitive that proteins of the nuclear envelope will participate in the movement and positioning of nuclei. With few exceptions in which nuclei are moved by bulk movement of the cytoplasm (Ramos-García et al., 2009), nuclear envelope proteins are required for the nucleus to interact with the cytoskeleton. In turn, the cytoskeleton provides the force to move nuclei, but requires specific and often highly regulated interactions with the nuclei (Gundersen and Worman, 2013). This is true in muscle also. Both the LINC complex (Linker of nucleoskeleton and cytoskeleton; reviewed Tapley and Starr, 2013), and the nucleoskeleton, which is a filamentous network of proteins that provides structure to the nucleus, are essential for nuclear movement and positioning in muscle cells. Moreover, mutations in several of these proteins have been identified in patients with muscle disease, specifically EDMD (Stewart et al., 2007).

## The LINC complex

The LINC complex is composed of Nesprin proteins (also known as Klarsicht, Anc, and Syne Homology (KASH) proteins) that span the outer nuclear envelope and SUN proteins that span the inner nuclear envelope. Nesprin proteins come in many isoforms. Mammals have at least four different Nesprin genes and each of these genes is differentially spliced to form in total dozens of Nesprin proteins. Similarly, SUN proteins exist in at least two different varieties from two different genes termed Sun1 and Sun2. The LINC complex and its general roles in nuclear positioning have been reviewed (Tapley and Starr, 2013), but we will focus here in greater detail on the data from muscle systems and its impact on muscle function.

Capitalizing on work in *C. elegans* (Starr et al., 2001; Starr and Han, 2002), the role of the Nesprin protein, Syne-1, was examined in mouse muscles. Expression of a dominant negative Syne-1 protein, which can localize to the nucleus but cannot interact with the cytoskeleton, displaced endogenous Syne-1 from the nucleus without generally disrupting nuclear structure. The disruption of endogenous Syne-1 localization did not appear to dramatically impact the peripheral localization of nuclei nor did it affect their general spacing. However, the clustering of nuclei at the NMJ was lost (Grady et al., 2005). Further analysis found that genetic deletion of the Syne-1 KASH domain, the domain that enables localization to the nuclear envelope, caused both synaptic and non-synaptic nuclei to be mispositioned (Zhang et al., 2007; Puckelwartz et al., 2009). Similarly, deletion of both SUN proteins, Sun-1 and Sun-2, resulted in fewer nuclei at the NMJ and the clustering of nuclei throughout the muscle fiber (Lei et al., 2009). Finally, although disruption of Syne-1 did not impact Sun-1 or Sun-2 localization (Grady et al., 2005), the deletion of Sun1/2 decreased the localization of Syne-1 to the nucleus. However, neither Sun1/2 deletion nor Syne-1/Syne-2 deletion impacted the organization of the nucleoskeleton (Lei et al., 2009). This indicates that the localization of proteins necessary for nuclear movement in muscle proceeds in a unidirectional manner from the nucleoplasm to the cytoskeleton.

The role of the LINC complex in positioning muscle nuclei is not confined to *in vivo* mouse muscles. The same proteins have been shown to be essential for moving nuclei in the mouse cell culture system of C2C12 myotubes. Specifically, it has been demonstrated that disruption of the LINC complex by expression of a dominant negative Syne-1 protein, similar to the experiment carried out *in vivo*, causes nuclei *in vitro* to move less dynamically and therefore to cluster (Wilson and Holzbaur, 2012). Similarly, in developing *Drosophila* larvae, deletion of the KASH domain from either of two KASH domain proteins in the genome (Klarsicht and Msp-300) results in clustered nuclei in the larval muscles (Elhanany-Tamir et al., 2012). Furthermore, mutation of the *Drosophila* SUN protein Klaroid, affected the position of nuclei in the embryonic musculature (Elhanany-Tamir et al., 2012).

The precise role of these LINC complex proteins during nuclear movement in muscles is not known. However, in a general sense, they enable the nucleus to interact with the cytoskeleton, which provides the force to move nuclei. For example in the C2C12 culture system, it has been demonstrated that KASH proteins enable the microtubule motors Kinesin-1 and cytoplasmic Dynein to interact with and move nuclei (Wilson and Holzbaur, 2012). This is consistent with data from several other systems including *C. elegans* (Meyerzon et al., 2009; Fridolfsson et al., 2010) and mammalian neurons (Zhang et al., 2009; Yu et al., 2011). But the data from *Drosophila* larval muscles suggest an alternative mechanism in which the KASH proteins are necessary to maintain microtubule-nucleus interactions (Elhanany-Tamir et al., 2012). Supporting this hypothesis, many KASH domain-containing proteins harbor domains that can directly interact with the cytoskeleton. However, despite the dramatic effect that the loss of KASH proteins have on microtubule organization, the effect could be indirect and result from inefficient recruitment of the aforementioned microtubule motors. Further work is necessary to distinguish these mechanisms and/or demonstrate how the two mechanisms are coordinated.

Another confounding issue in these data is that the initial study in mouse, in which Syne-1 and Syne-2 were displaced from the nuclear envelope by the expression of the Syne-2 KASH domain, only affected the positioning of the synaptic nuclei. It is not clear why the displacement of the endogenous protein from the nuclear envelope causes a different phenotype than does the expression of a KASH-less protein. A simple interpretation of these data is that a portion of the endogenous protein remains localized to the nucleus even in the presence of the dominant negative, and that the synaptic nuclei are more sensitive to levels of endogenous Syne-1 and Syne-2. However, further work is necessary to fully understand these data.

## The nucleoskeleton

The nucleoskeleton is a meshwork of proteins contained within the nucleus and adjacent to the inner nuclear membrane that provides the nucleus with its shape and its ability to withstand mechanical stresses. The primary components of the nucleoskeleton are the nuclear lamin proteins which exist in several varieties. There are two B-type lamins that originate from two genes, *LMNB1* and *LMNB2*. The A-type lamins, Lamin A and Lamin C, are, respectively, the immature and fully processed gene products of the *LMNA* gene and will be the forms discussed here; it is these proteins that directly contribute to nuclear positioning in muscles, and mutations in the *LMNA* gene result in the autosomal dominant form of EDMD (Stewart et al., 2007).

Work in cell culture has demonstrated that in the absence of Lamin A/C, nuclear movement is inhibited (Lee et al., 2007; Hale et al., 2008; Houben et al., 2009; Folker et al., 2011), the ability of the nucleus to withstand physical stress is limited (Broers et al., 2004; Lammerding et al., 2004), and the ability of the cell to organize its genome is compromised (Gnocchi et al., 2011; Mattout et al., 2011). Each of these biological functions has been, and continues to be, explored as possible pathogenic mechanisms of *LMNA* mutations and significant data support each of these hypotheses.

The first *Lmna*^−/−^ mouse study was published in 1999 and changes in both nuclear structure and nuclear localization were noted. Moreover, mice lacking Lamin A/C were described as dystrophic (Sullivan et al., 1999). All of these characteristics were similar to those described in human EDMD patients carrying *LMNA* mutations (Bonne et al., 2000). Similarly, larval muscles in *Drosophila* which lack Lamin C (the only A-type lamin in *Drosophila*) have nuclei with variable and distorted structures that are commonly mispositioned (Dialynas et al., 2010; Zwerger et al., 2013). Yet, none of these studies have been able to clarify the relative contributions of distorted nuclear structure and aberrant nuclear positions to muscle disease.

Attempts to clarify this question using cell culture based systems have added support for each possibility. For example, more detailed rheological analysis has clearly demonstrated that not only does the loss of Lamin A/C make cells and their nuclei more sensitive to mechanical stress, but that mutations which cause EDMD have the same effect (Zwerger et al., 2013). Similarly, *LMNA* mutations that when heterozygous in humans cause EDMD inhibit nuclear movement when expressed in fibroblasts suggesting a dominant negative role for these mutations. Interestingly, *LMNA* mutations that cause Dunnigan Type 2 Familial-Partial Lipodystrophy, also in a dominant negative manner, have no effect on nuclear movement, suggesting that mediating nuclear positioning or nuclear-cytoskeletal interactions are a function of Lamin A/C that is particularly important in muscle (Folker et al., 2011).

Finally, although the experiments in *Drosophila* do not differentiate between effects on nuclear structure, gene regulation, and nuclear position, they do provide insight toward the relevance of nuclear position. As noted previously, the consensus has been that the mispositioned nuclei in patients with muscle disease are merely a result of ongoing myofiber repair. However, there is no evidence that *Drosophila* larval muscles undergo repair. Yet, the nuclei in *Drosophila* larval muscles are dramatically mispositioned when Lamin C is absent or when disease causing variants of Lamin C are expressed only in the muscle (Dialynas et al., 2010). This suggests that myonuclear positioning is an active and critically maintained process and that all nuclear mispositioning is not merely a marker of ongoing muscle repair.

Taken together, these data make clear that Lamin A/C is essential for proper nuclear positioning in muscle. Additionally, and most importantly for this discussion, is that the contribution of Lamin A/C to nuclear position is inhibited by mutations that cause muscle disease. This correlation suggests that the role of Lamin A/C in positioning nuclei may contribute to muscle weakness and disease. More generally, these data further suggest that the positioning of the nucleus within the muscle may be fundamentally important and that aberrant nuclear positioning may contribute to disease pathogenesis.

Proteins that interact with the Lamin A/C also cause muscle disease and have also been implicated in regulating nuclear structure, gene expression and nuclear position (Zhong et al., 2010). Emerin (*EMD*) is among the best described Lamin-interacting proteins; it was identified as a gene mutated in patients with X-linked EDMD prior to the identification of *LMNA* as the gene responsible for the autosomal dominant form of EDMD (Bione et al., 1994). Emerin null fibroblasts are similar to Lamin null fibroblasts in that they fail polarize and instead form inefficient nucleus-cytoskeleton interactions (Chang et al., 2013; Ho et al., 2013). However, the analysis of Emerin and its functions *in vivo* are limited when compared to Lamin A/C. Analysis of the Emerin null mouse has likely lagged relative to the Lamin null mouse due to the lack of phenotype. Although the Emerin null mouse does have delayed muscle regeneration (Melcon et al., 2006), there are no overt dystrophic phenotypes (Melcon et al., 2006; Ozawa et al., 2006). The reason for this discrepancy requires further examination, but perhaps Emerin is involved in enhancing or specifying a specific Lamin A/C function. If Lamin A/C is contributing to muscle function through multiple pathways, one might reason that the effects of mutating each individual regulating protein would be diminished relative to loss of Lamin A/C itself.

Unfortunately, it is not clear how mutations in *EMD* and *LMNA* cause muscle disease. However, both genes are necessary to maintain the structure of individual nuclei, to position nuclei, and to maintain proper gene regulation as discussed above. Perhaps these three aspects of nuclear biology in muscle are critically linked.

Indeed, it has been argued that improper gene regulation in *Lmna* null mice causes the clustering of nuclei. This clustering is particularly evident near the NMJ, and nuclei in this location vary from levels of almost no acetylated histone H3 to high levels of acetylated histone H3. This is contrasted by the nuclei in WT muscles which have consistent and moderate levels of acetylated histone H3 (Gnocchi et al., 2011). However, it is equally plausible that improper positioning leads to the change in gene expression. There is in fact clear evidence that nuclear position can influence gene expression. For example, nuclei at the NMJ have a unique transcriptional profile relative to the non-synaptic nuclei (Jevsek et al., 2006). Perhaps nuclei being in close proximity can communicate and coordinate their transcriptional output such that individual nuclei down-regulate transcription. Alternatively, nuclei may sense the proximity of other nuclei and up-regulate transcription in an effort to repair or remodel the muscle. Although the cause-effect relationship is not clear, that both phenotypes are common and can be caused by mutations in the nucleoskeleton highlights the need to better understand how these processes relate to muscle function. The ability to affect the position of nuclei without directly affecting their transcriptional profile, and vice versa, is essential to gaining a full understanding of this relationship.

## The cytoskeleton

Movement of nuclei by the cytoskeleton is seen in eukaryotes ranging from yeast to mammals and is relevant to processes ranging from DNA segregation during mitosis to cellular locomotion (Gundersen and Worman, 2013). In the next several paragraphs we will consider how the cytoskeleton moves nuclei and will focus on mechanisms determined in muscle systems.

Two different cytoskeletal networks have been demonstrated to drive nuclear movements. Most nuclear movements, in both muscles and other tissues, are driven by microtubules and their associated proteins and motors. Other nuclear movements and positioning events require the action of the actin cytoskeleton and its associated factors. In most cellular contexts the actin network and the microtubule network are intimately connected, often co-regulated, and can directly impinge on the activity of the other, making it difficult to discern the specific effects of either network (Rodriguez et al., 2003). Still, several mechanisms of either nuclear movement or nuclear positioning have been elucidated and attributed to one cytoskeletal network or the other.

## Microtubules

The organization of the microtubule network in muscle cells is different from that in most other cell types. Most eukaryotic cells have a single microtubule organizing center (MTOC) from which most microtubules emanate and at which microtubule minus-ends are anchored. In higher eukaryotes this is accomplished by the centrosome and in many lower eukaryotes such as yeast, this is accomplished by an analogous structure called the spindle pole body. Muscle cells do not have a single MTOC. This is not merely a result of having many nuclei because each nucleus has several associated MTOCs. In culture, after myoblasts fuse to a growing myotube they disassemble their centrosome and redistribute their pericentriolar material and γ-tubulin around the entire nuclear envelope (Tassin et al., 1985) and in smaller quantities to the Golgi apparatus (Ralston et al., 2001). Similar organization is seen *in vivo*, where each nuclear envelope and Golgi apparatus thus serves as a MTOC with microtubules emanating from many locations on both the nucleus and the Golgi apparatus (Oddoux et al., 2013). Given that there are often tens to hundreds of nuclei in a given muscle, mature muscles have microtubules that originate from many distinct locations.

Except for the number of MTOCs, the microtubules emanating from the nuclei behave similarly to those in other cell types. Microtubules grow in all directions with equal probabilities and have similar dynamics to microtubules in standard cell culture experiments (Wilson and Holzbaur, 2012) and *in vivo* (Oddoux et al., 2013; Folker et al., 2014). However, this is not the only microtubule network in muscle cells. In the mature muscles of mammals and flies, a second microtubule network is present within the myofibril network and is characterized by a significantly different population of microtubules. Microtubules in this region are less dense and are oriented such that they run along the length of the myofibrils, with occasional microtubules running transversely between the myofibrils (Kano et al., 1991; Metzger et al., 2012). Additionally, it appears that many of the microtubules that exist in this central portion of the muscle originate from perinuclear regions near the muscle periphery (Kano et al., 1991). Thus, it is likely that the nuclei serve as the MTOC for both microtubule networks that are observed within skeletal muscle. Furthermore, given that microtubules are directly interacting with nuclei in muscles, the organization and activity of the microtubule cytoskeleton will inevitably impact the spatial distribution of nuclei.

The role of microtubules in positioning muscle nuclei dates back to early studies using explants from chick embryos which demonstrated that nuclei moved, rotated and eventually became fixed in position (Capers, 1960). Subsequent analysis using cultures derived from mice and rats found that nuclei underwent similar movements and further demonstrated that the dynamic movements required microtubules. Specifically, it was shown that if microtubules were depolymerized with colchicine, nuclear movements and rotations stopped (Englander and Rubin, 1987).

Remarkably, little more was learned regarding how the microtubule cytoskeleton moves muscle nuclei until recently. New work has confirmed a role for microtubules in moving muscle nuclei and expanded the mechanistic understanding of the process. Generally, the proteins that move nuclei in other systems (Gundersen and Worman, 2013) contribute to the movement of nuclei in muscle systems.

The two factors that generate most of the force that moves nuclei in muscles are the two microtubule motors, Kinesin-1 that moves toward microtubule plus-ends, and cytoplasmic Dynein that moves toward microtubule minus-ends. These two motors are also essential for microtubule based nuclear movement in virtually every other system (Tapley and Starr, 2013), suggesting that the basic mechanisms are conserved among cell types and species. However, there are several unique aspects to nuclear movements in muscle. Furthermore, recent analyses have described distinct mechanisms that contribute to different types of nuclear movements in muscle both *in vivo* during embryonic *Drosophila* development and in mouse culture systems (Cadot et al., 2012; Folker et al., 2012; Metzger et al., 2012; Wilson and Holzbaur, 2012).

One of the most striking aspects of nuclear movement in muscle is that the nuclei dynamically rotate in three dimensions during translocation. This aspect was also first noted in cultures derived from chick embryos (Capers, 1960) but has recently been described in mammalian culture systems (Wilson and Holzbaur, 2012) and developing *Drosophila* embryos (Folker et al., 2014). Furthermore, moving myonuclei in the developing *Drosophila* embryo have a defined leading and lagging edge which enables rapid changes in nuclear shape. These shape changes require the coordinated actions of Kinesin and Dynein at the nucleus, an aspect of nuclear movement that has to date only been described in developing muscle (Folker et al., 2014).

The role of these rotations and shape changes are not clear. However, each of these reports hypothesizes that these behaviors provide nuclei with a unique ability to maximize movement velocity in dense cellular and embryonic environments. Similar rotations of translocating nuclei have been noted in *C. elegans* where rotations were also proposed as a means to navigate the dense cellular environment (Fridolfsson and Starr, 2010). Additionally, dramatic changes in nuclear shape have been noted in neurons (Tsai et al., 2007) where they seem to be essential to move through spatially restricted environments.

Although similar behaviors have been noted in other systems, the mechanisms and persistence of these behaviors in muscle are different. For example, the nuclear rotations in *C. elegans* appear to occur only to navigate past blockages whereas in muscle, nuclear rotations are common and are not strictly correlated with defined translocation (Wilson and Holzbaur, 2012; Folker et al., 2014). Additionally, the changes in nuclear shape during translocation in neurons are dependent only on the activity of Dynein from a position distant from the nucleus (Tsai et al., 2007), whereas the analogous behavior in muscle requires the spatially segregated activities of Dynein and Kinesin (Folker et al., 2014). These distinctions may be driven by the multinucleate nature of muscle and may reveal information regarding interactions between nuclei. If nuclei do indeed interact with one another, it is likely that nuclear position affects these interactions. Altered interactions between nuclei could greatly influence the maintenance of myodomains as well as the transcriptional profile of individual nuclei, and thus have dramatic effects on muscle structure and function.

Kinesin and Dynein move nuclei in muscle systems but they contribute to different types of movement using different arrays of regulators/accessory proteins. Consider again the types of nuclear movement in the muscle. In simple terms, there is (1) movement to the center of the myotube/myofiber following fusion, (2) movement of each nucleus to the muscle periphery, (3) equidistant spacing of nuclei, and (4) movement of nuclei to the NMJ. Experiments using mouse culture systems have identified the small GTPase Cdc42, and the polarity proteins Par6 and Par3, as necessary for newly fused nuclei to move toward the center of the myotube (Cadot et al., 2012). Each of these proteins contributes to nuclear movement in other systems by enabling Dynein anchored at the cell cortex to pull nuclei that are attached to microtubule minus-ends toward itself (Kotak and Gönczy, 2013). Nuclei in muscle are moved by a similar mechanism, but the details may be slightly different. In immature myotubes, Dynein, Par3, and Par6 localize to the already incorporated nuclei. From the central cluster of nuclei, Dynein pulls the new nuclei to the myotube center (Cadot et al., 2012). *In vivo* experiments looking at embryonic muscle development in *Drosophila* suggest mechanisms more analgous to those in *C. elegans*. Specifically, Dynein is anchored at the muscle cortex by Pins and pulls microtubule minus-ends and the attached myonuclei toward the end of the muscle dependent on the microtubule plus-end tracking protein, CLIP-190 (Folker et al., 2012). The difference between the data in mammalian cell culture and that in developing *Drosophila* embryos may result from *in vitro/in vivo* differences or because different types of nuclear movement are being analyzed. That other mechanisms seem to be conserved between the two systems suggests that the latter may be the case.

The study of nuclear movement in muscle has revealed novel behaviors of moving nuclei (Wilson and Holzbaur, 2012; Folker et al., 2014), and has also identified proteins with novel roles in nuclear movement. MAP7/Ensconsin was long ago identified as a microtubule associated protein (Bulinski and Bossler, 1994), but a cellular role for this protein had not been identified. Work in both the developing muscles of the *Drosophila* embryo and mammalian cell culture have found MAP7/Ensconsin to be essential for nuclear movement in muscle (Metzger et al., 2012). Additionally, unlike Cdc42, Par6, Par3, and Dynein, MAP7/Ensconsin does not affect the movement of nuclei toward the muscle center, but is essential only for the spacing of nuclei throughout the muscle by a mechanism identified in both developing *Drosophila* and mammalian culture systems further illustrating that different types of nuclear movement in muscle are driven by distinct mechanisms (Cadot et al., 2012; Metzger et al., 2012). The mechanism by which MAP7 contributes to nuclear movement is not known. However, MAP7 can physically interact with Kinesin (Metzger et al., 2012), and the *Drosophila* homolog of MAP7, Ensconsin, can increase Kinesin-microtubule interactions, thus resulting in increased Kinesin motility (Sung et al., 2008). Finally, a fusion protein containing the MAP7 microtubule binding domain and the Kinesin motor domain can move nuclei (Metzger et al., 2012). These data have all been used to suggest that MAP7/Ensconsin helps spread and maintain the spacing between nuclei by enabling Kinesin to slide antiparallel microtubules which emanate from neighboring nuclei, similar to the way in which Kinesin and Ensconsin transport microtubules in neurons (Barlan et al., 2013). The result of this sliding is the pushing apart of adjacent nuclei similar to the mechanism by which mitotic spindles are elongated in cell divisions (Metzger et al., 2012).

To date, mutations in Dynein and its regulatory proteins, Kinesin and its regulatory proteins, and MAP7/Ensconsin have not been identified in patients with muscle disease. That is likely due to the very fundamental roles each of these proteins play in all cells. Thus, if the ability of Dynein and/or Kinesin to move nuclei is eliminated, its ability to move other cargos throughout the cell are also likely compromised. However, these analyses have provided insight to the relevance of nuclear positioning in muscle. Tissue specific depletions of these proteins in *Drosophila* have confirmed that these proteins have a muscle autonomous effect on nuclear positioning without affects on nuclear morphology (Folker et al., 2012; Metzger et al., 2012). Yet, the ability of *Drosophila* lacking these proteins specifically in the muscle to move is inhibited (Folker et al., 2012; Metzger et al., 2012). This is not to suggest that nuclear morphology and gene regulation are not essential and relevant contributions to disease. Instead these data makes evident that the clustering of nuclei, in the absence of other obvious defects in muscle architecture, does inhibit muscle function.

## Actin

There are far fewer examples of actin-dependent nuclear movement compared to microtubule-dependent nuclear movement throughout biology. Furthermore, there is no evidence of actin-dependent nuclear movement in muscle. However, there is evidence that actin contributes to the anchoring of nuclei in different locations (Zhang et al., 2002, 2010; Puckelwartz et al., 2009). Additionally, there is substantial evidence from experiments in cell culture that nuclear proteins interact with actin and that these interactions can influence nuclear structure (Nikolova et al., 2004; Lüke et al., 2008; Khatau et al., 2009), cellular rheology (Maniotis et al., 1997; Lammerding et al., 2004), and nuclear movement and positioning (Luxton et al., 2010).

In fibroblasts, actin moves the nucleus as an initial step in cell migration (Gomes et al., 2005). Furthermore, this movement requires the same LINC complex components that are mutated in patients with muscle disease. As in muscle, the LINC complex enables the direct interaction between the nucleus and the cytoskeleton, but in this case the nucleus interacts with the actin cytoskeleton rather than the microtubule cytoskeleton (Luxton et al., 2010). Similarly, Lamin A/C is necessary for nuclear movement in this system and contributes by serving as an anchor for the LINC complex so that it can couple the movement of actin to the nucleus. Essential to this review, mutations in Lamin A/C that cause muscle disease also inhibit the ability of the nuclear lamina to anchor the LINC complex (Folker et al., 2011). This raises the possibility that the ability of Lamin A/C to anchor the LINC complex so that force can be transmitted from the cytoskeleton to the nucleus is fundamental to muscle biology and muscle disease pathogenesis.

Only one report has suggested even indirect roles for actin in regulating the position of myonuclei *in vivo*. It was demonstrated that the KASH domain containing protein, Msp-300, was essential for nuclear positioning in larval muscles. Although most of this work focused on the effects that the loss of Msp-300 had on the organization of microtubules, it also found Msp-300 to be localized to the Z-disks suggesting a role in sarcomere organization (Elhanany-Tamir et al., 2012). Furthermore, although Msp-300 did not interact directly with actin, it did interact with actin via the thick filament protein, Titin and these interactions may be necessary for proper nuclear positioning.

Although there is limited evidence for actin dependent nuclear movement in muscle, the fact that genes identified as causes of EDMD are essential for actin-dependent nuclear movement in other systems is compelling. Furthermore, it has been reported that mutations in each of these genes in addition to having effects on the nucleus as discussed throughout this review, also affect actin organization (Ho et al., 2013). And work in *Drosophila* and mice has found that the genetic disruptions that cause nuclear mispositioning (along with other effects) also impact the organization of the actin cytoskeleton (Dialynas et al., 2010). Thus, despite far less evidence for actin dependent nuclear movement, further exploration of this possibility is necessary.

## Conclusion

The subcellular structure and organization of muscle has been studied since the advent of microscopes. Although, the assembly and organization of myofibrils which dominated early research is still being examined, new avenues of research have emerged. In general, the questions of where the different organelles are located, why they are located in such a manner, how they become localized, and whether the organization of different organelles are linked have garnered increased focus. Yet, the complex organization of individual muscle cells, the multinucleate nature of individual muscle cells, and the bundling and further bundling of these cells have provided many obstacles to detailed understanding of muscle development.

Nevertheless the technology and systems to address these questions are becoming available (Oddoux et al., 2013). Although this review focused on how nuclei move and the correlations between nuclear positioning and muscle disease, similar analyses have been performed with respect to mitochondria (Pathi et al., 2012), t-tubules (Flucher et al., 1994) and other organelles. We have highlighted some of the data regarding the mechanisms of nuclear movement in muscle and indicated that the basic principles of nuclear movement are conserved between species and between cell types. The conservation of the proteins used to move nuclei provides a list of proteins to examine in systems of muscle development. Furthermore, it expands the list of targets that we should evaluate in patients suffering from muscle disease.

Indeed, many of the proteins that are necessary to move nuclei are mutated in individuals with muscle disease. However, this is almost exclusively true of those proteins that localize to the nucleus and contribute from that location by regulating the interactions between the nucleus and the cytoskeleton. The cytoskeletal proteins that contribute to nuclear movement in muscle have not yet been linked to muscle disease. This is likely because mutations that would affect the ability of the cytoskeleton to move nuclei would also cause general developmental defects as has been demonstrated for Kinesin (Wang et al., 2013). But it is important that the contribution of these proteins to nuclear movement not be ignored on grounds that they do not cause disease. With regards to basic biology, these genes can provide a means to study nuclear position in the absence of global effects on nuclear architecture and gene regulation. More therapeutically relevant, they are essential for a process that is highly correlated with disease. Thus, with sufficient understanding it may be possible to circumvent the disease causing mutations by targeting the cytoskeleton.

Despite the high correlation between aberrant nuclear positioning and muscle disease the idea that nuclear position in muscle is essential for muscle function will likely remain controversial. Recent analyses in *Drosophila* which demonstrated reduced muscle output when nuclei were mispositioned without additional underlying defects (Metzger et al., 2012) may convince some, but not all. However, reconsidering the process of muscle repair may provide the most compelling evidence that nuclear movement is important and essential, even if mispositioned nuclei do not cause disease. Organisms, and cells, in general optimize their energy usage. With that premise, it is unlikely that nuclei would move to the center and then back out to the periphery of an already mature myofiber. Energetically speaking it would be far more efficient to incorporate a new nucleus at the point of entry at which point the nuclei could undergo slight movements to space along the myofiber. Nuclear movement to the center and then back to the periphery of a muscle must be essential to muscle development and repair. With newly found focus we may soon understand the biological necessity of these long range nuclear movements in muscle.

Finally, nuclear position is almost certainly not the final answer with regards to muscle disease. But with the evidence that nuclear positioning is essential to muscle function is increased, making it time that the muscle biology community begin to consider centrally localized nuclei as more than merely a marker of ongoing muscle repair and as a phenotype that may influence muscle function and health.

### Conflict of interest statement

The authors declare that the research was conducted in the absence of any commercial or financial relationships that could be construed as a potential conflict of interest.
